# Male Triple-Negative Breast Cancer

**DOI:** 10.7759/cureus.14542

**Published:** 2021-04-18

**Authors:** Qasif Qavi, Firas Alkistawi, Shashi Kumar, Rizwan Ahmed, Abdalla Saad Abdalla Al-Zawi

**Affiliations:** 1 Surgery, Basildon and Thurrock University Hospital, Basildon, GBR; 2 Surgery, Mid and South Essex NHS Foundation Trust, Basildon, GBR; 3 Surgery, Princess Royal University Hospital, Orpington, GBR; 4 Breast Surgery, Anglia Ruskin University, Chelmsford, GBR; 5 Breast Surgery, Basildon and Thurrock University Hospital, Basildon, GBR; 6 General Surgery, Mid and South Essex NHS Foundation Trust, Basildon, GBR

**Keywords:** male breast cancer, triple negative breast cancer, mastectomy, radiotherapy, klinefelter syndrome

## Abstract

Male breast cancer is a rarely encountered disease, when compared with female breast cancer, often detected in more advanced stage at the time of diagnosis, and associated with more lymph node metastasis rates, more estrogen receptors positivity, and less human epidermal growth factor receptor-2 expression (HER-2) rates. Surgical management also shows some difference, where the most common operative technique of male breast cancer patients is mastectomy and/or axillary surgery. Triple-negative breast cancer is less frequent than other subtypes and is associated with poorer prognosis. This is because of its association with higher histopathological grade than that in other types of breast cancer. Only fewer treatment options are available compared to hormone-positive, HER-2 positive breast cancer. We are present a case of 71-year-old gentleman with triple-negative breast cancer.

## Introduction

Breast cancer in men is relatively uncommon, but its incidence has been rising. A male’s lifetime risk of having breast cancer is around 1 in 1,000 [1]; however, the incidence is higher as the age is closer to the seventh decade of life. Male breast cancer (MBC) is a rare entity, representing approximately 1% of all male different cancers and approximately 1% of all both genders breast cancer worldwide [2]. It has also been shown that most MBCs (95%) are hormone receptor positive, which is higher than that of female breast cancer [3]. Patients with MBC have a worse prognosis compared with female breast cancer because of more advanced disease and older age at presentation [4]. We present a rare case of triple-negative breast cancer subtype, which is considered to be more aggressive and associated with poorer prognosis than other subtypes of breast cancer. There is a real challenge in dealing with such a cancer variety because of the fewer treatment options available for such a case.

## Case presentation

A 71-year-old man presented with a right breast lump that was noticed three weeks prior to the clinic appointment. His background history included gastroesophageal reflux, chronic kidney disease, repair of right inguinal hernia, and excision of basal cell carcinoma of the abdominal wall. Clinical examination revealed a dent in the skin and a palpable 3-cm lump in the axillary tail.

**Figure 1:**
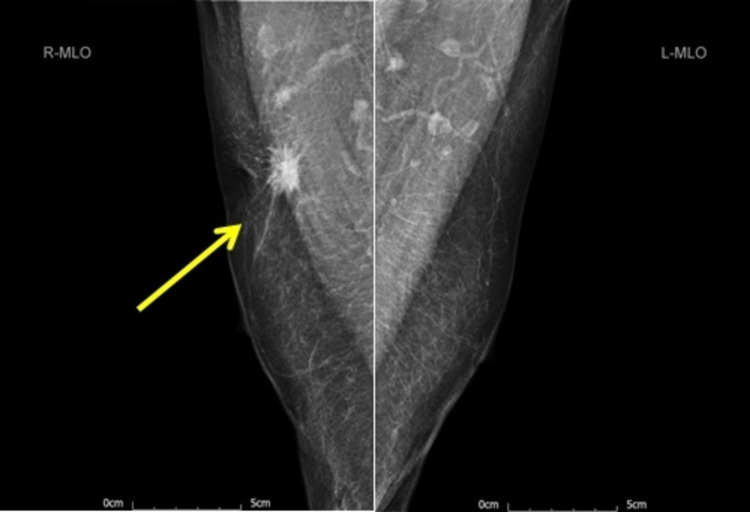
Bilateral mammogram (mediolateral oblique view) showing a 25-mm suspicious lesion in the right breast axillary tail (yellow arrow).

**Figure 2:**
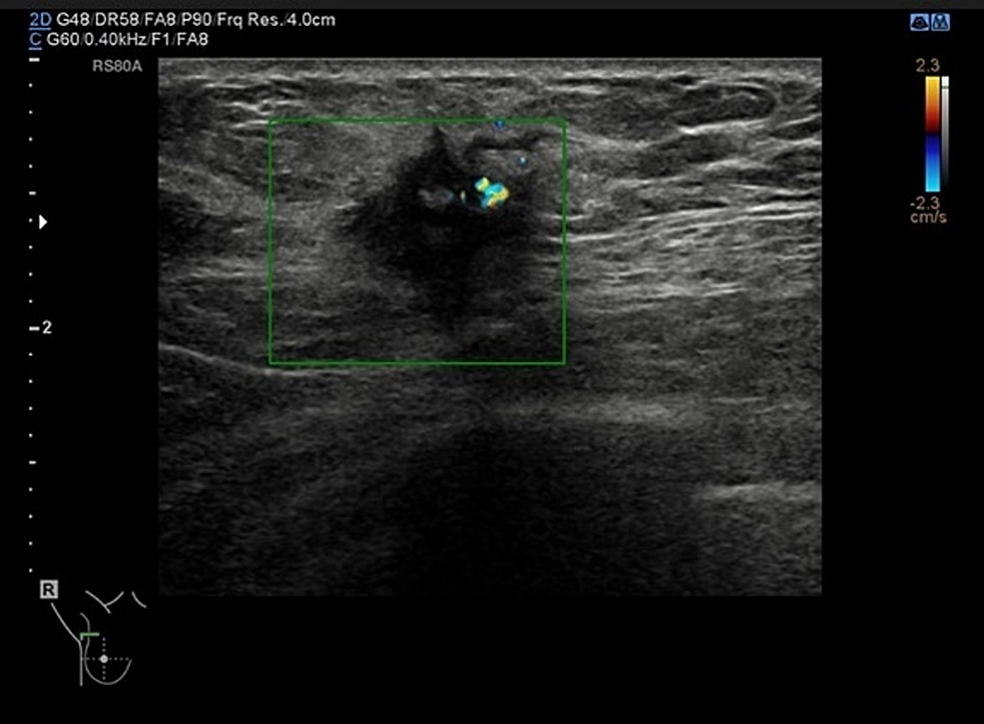
Right breast ultrasound showing a 25-mm suspicious lesion in the right breast axillary tail (green square).

**Figure 3:**
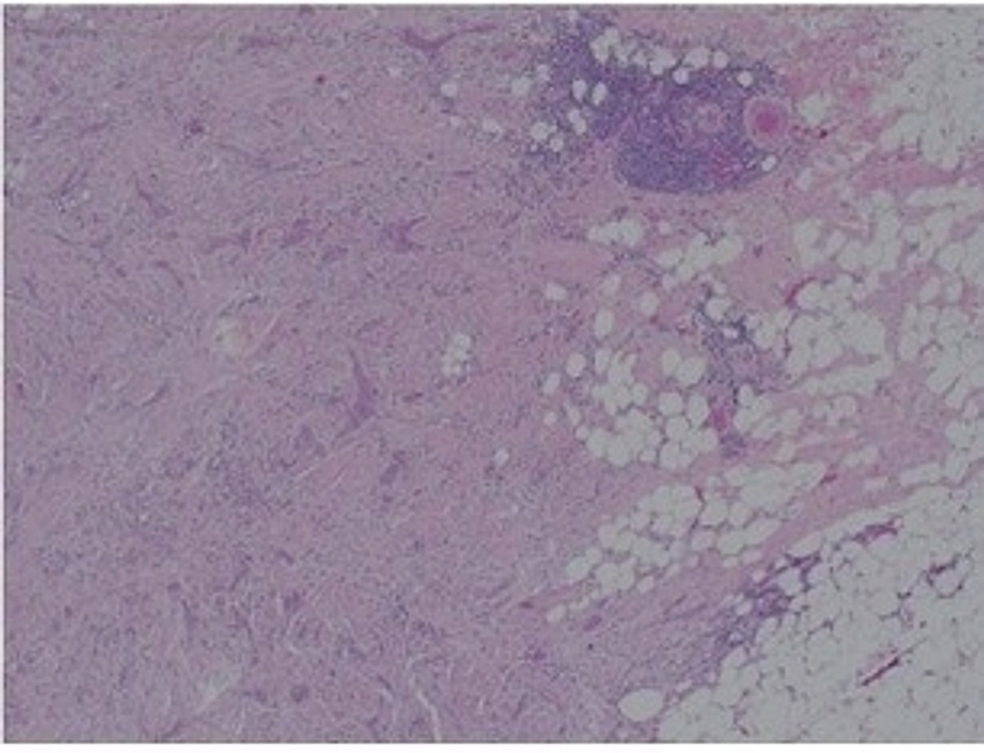
H&E (x10) sections of the breast tissue (after mastectomy) showing irregular cords of carcinoma in densely fibrotic stromal background.

Mammogram (Figure 1) showed a 25-mm suspicious lesion in the right axillary tail, which was also visualized on ultrasound (Figure 2), along with a suspicious lymph node in the right axilla. Imaging-guided core biopsies were taken from both; the histopathology was consistent with grade 1 invasive ductal carcinoma, and ER 0, PR 0, and HER 2 negative as well. Mastectomy with axillary clearance was performed after consultation with breast MDT (multidisciplinary team). The post-operative histopathology revealed 23-mm, grade I, triple-negative invasive ductal carcinoma (Figure 3) with lymphovascular invasion, Ki67 10%, one out of nine lymph nodes showing metastatic disease, and the final staging as T2N1M0. The MDT advised for annual surveillance for five years.

## Discussion

Breast cancer is the commonest diagnosed women cancer globally and considered as the leading cause of cancer-related mortality in females [5].

Breast cancer in males is very rare, accounting for only less than 1% of all malignant diseases in men [1], and forms around 1% of all breast cancer cases detected in both genders.. The current observations revealed that its incidence is increasing [3]. Breast cancer in both genders shares common features; however, still there are remarkable dissimilarities in age at diagnosis, incidence rates, treatment, prognosis, and survival. Males are diagnosed with breast cancer at an average of 10 years older than the age at which it is diagnosed in women of 65 years; however, it has been reported that it has been diagnosed in a 17-year-old boy as well [6]. Risk factors associated with increased risk of MBC (Table 1) are family history, cryptorchidism, orchidectomy, orchitis, infertility, Klinefelter’s syndrome, smoking, physical inactivity, alcohol consumption, previous thoracic radiotherapy, altered estrogen-testosterone ratio, and use of exogenous androgens and estrogen, and it has been diagnosed in male‐to‐female transgender as well [1,7-9]. It is also becoming clearer that obesity and the associated metabolic abnormalities may play an important role in the development of MBC as a result of decrease in androgen level and increase in estrogen level [3].

**Table 1:** Risk factors for male breast cancer [5,8,9]

Family history
BRCA1/2 mutation
Use of external androgens and estrogen
High BMI
Sedentary lifestyle
Smoking
Radiation exposure: medical/occupational
Alcohol consumption
Klinefelter syndrome
Cryptorchidism
Orchidectomy
Orchitis
Infertility
Liver cirrhosis

The breast cancer risk in females with BRCA1 or BRCA2 mutation is estimated at 90% [10]; the two gene mutations are not only responsible for 90% of familial breast cancer but also for the most of the familial ovarian malignancies [11]. Only one-third of males who are BRCA1/2 mutation carriers will develop cancers as breast, prostate, and pancreatic malignancies. With BRCA2 mutation being more common in MBCs, BRCA2 mutations are thought to be found in up to 14-40% of MBC cases. It is recommended that male patients with breast cancer to be referred for genetic counselling and tested for BRCA gene mutation [3,8,11]. MBC clinically it presents as lump, pain, or skin dent. Occult disease is extremely rare, and the disease is identified as an occult disease when a patient presents with axillary node metastases without obvious or an identifiable breast primary. Triple-negative MBC (TNMBC) could be associated with other conditions such as dermatomyositis [8,12]. The commonest histological subtype of MBC is ductal carcinoma of no special type; thus, our case falls in this category. Around 95% of the invasive MBCs are luminal A or B. Other subtypes such as papillary carcinoma and Paget disease have also been reported [3,13].

MBC clinically presents as a lump, pain, or skin dent; occult disease is extremely rare and may be detected as axillary node metastases without obvious or an identifiable breast primary [8]. Most of the diagnosed breast cancers in both genders show estrogen receptor expression; however, the positive hormone receptor expression rate in MBC is greater than that in women breast cancer, that is, up to 95% [3,12]. Around 10-15% of the newly diagnosed breast cancer cases are triple-negative disease cancer, which is generally known to be an aggressive subtype [14].

The Ki-67 proliferative index, which is used as a prognostic and predictive tool in breast cancer [15], is reported to be highly expressed in 20-40% of MBC in general.

Chavez-Macgregor et al. in a cohort of 606 MBC patients reported that metastatic TNMBC) is found in only 3.6%, where 15% are Her-2 enriched tumors and 81% are hormone-sensitive. TNMBC is an aggressive disease subtype, frequently diagnosed at a later stage, associated with larger tumor size, a higher tumor grade, poorly differentiated histological subtype, and increased rate of lymph node metastasis, and detected more at a younger age [16].

TNMBC has a significantly higher rate of recurrence and mortality compared with hormone-positive breast cancer [14,16], and it also has a notably poorer prognosis than female TNBC or other breast cancer subtypes [17]. Even though some authors argue that these tumors respond to chemotherapy better than other subtypes of invasive breast cancer, their prognosis remains poor. The standard treatment for MBC follows the same pathway as that of female breast cancer and depends on the stage of disease at diagnosis and hormone receptor status of the cancer. Mastectomy is regarded as the standard treatment for MBC due to the less amount of male breast tissue and debilitating side effects of adjuvant therapy [18]. Contrary to female breast cancer, breast conservation surgery does not play a role in the management of MBC due to the small volume of breast tissue in men. As triple-negative breast cancer responds better to chemotherapy than hormone receptor positive cancers [12], and around 80% (majority) of TNBC cases receive systemic chemotherapy [19]. Many reports showed that adjuvant chemotherapy is indicated for MBC cases with poor prognostic clinical and biological indicators, and this group had almost the same disease-free survival when compared with patients who had less aggressive disease and had no chemotherapy. This indicates that chemotherapy may have a survival benefit for the high-risk group of MBC. Referring to our case, few of the chemotherapeutic drugs are excreted through the renal system, and the use of these drugs in chronic kidney disease patients could be a real challenge for the physician, as it could be difficult to achieve a safe therapeutic level without exposure to drug toxicity. The prognostic markers in our case, such as small tumor size, low histopathological grade, low Ki-67, N1 disease, and PREDICT tool with a chemotherapy benefit of only 2.4%, in addition to chronic kidney disease with the potential side effects, were not in favor of chemotherapy despite that the tumor is a triple negative disease with lymphovascular invasion.

The current data suggest that adjuvant radiotherapy has an advantageous effect as it improves the overall survival but not the cause-specific survival [20]. Generally, the unfavorable overall outcomes in MBC are attributed to the older age, late presentation, and advanced tumor stage at the time of diagnosis [4].

## Conclusions

We presented the management of a rare case of node-positive triple-negative breast cancer in a 71-year-old man treated with mastectomy and axillary node clearance.

Adjuvant chemotherapy was not offered due to the small tumor size, less aggressive disease, and debilitating side effects associated with chemotherapy. Not much is known about MBC, especially TNMBC, and as MBC detection rate is increasing and is often detected at an advanced stage, more clinical research is needed to guide clinical diagnosis and management. In addition, risk groups screening, public awareness, and encouraging males for self-examination are recommended to avoid delays in diagnosis.
